# Ace Your Self-Study: A Mobile Application to Support Self-Regulated Learning

**DOI:** 10.3389/fpsyg.2022.793042

**Published:** 2022-05-03

**Authors:** Martine Baars, Farshida Zafar, Micah Hrehovcsik, Edwin de Jongh, Fred Paas

**Affiliations:** ^1^Department of Psychology, Education, and Child Studies, Erasmus University Rotterdam, Rotterdam, Netherlands; ^2^Erasmus School of Law, Erasmus University Rotterdam, Rotterdam, Netherlands; ^3^Innovation Studio, HKU University of the Arts Utrecht, Utrecht, Netherlands; ^4^Dev66, Den Haag, Netherlands; ^5^School of Education/Early Start, University of Wollongong, Wollongong, NSW, Australia

**Keywords:** self-regulated learning, mobile application, m-learning, metacognitive strategies, cognitive strategies

## Abstract

Without guidance, students typically overestimate their understanding and memory of learning materials, which can have detrimental effects on the learning process. However, most students do not receive guidance or instruction about how to study. Moreover, students are largely unaware of strategies to self-regulate their learning and study effectively. Research has shown that prompting both cognitive and metacognitive strategies is effective to support self-regulated learning (SRL). Therefore we developed a mobile application, the Ace your self-study app, to prompt both cognitive and metacognitive strategies to support learning processes. In this article a theoretical background, description of the app’s features and design choices are presented. Also, data from the application in presented to give provide an idea of how the app has been used.

## Introduction

Self-regulation is an important skill in many domains of life. For example, to fight addiction (Baumeister and Vonasch, 2015), to remediate weight problems (Johnson et al., 2012), or to excel in athletics (Cleary and Zimmerman, 2001). Self-regulation in an academic setting, could be defined as self-regulated learning (SRL), and refers to the interaction of cognitive, motivational and contextual factors that promote academic achievements (e.g., Dinsmore et al., 2008; Schunk, 2008; Dent and Koenka, 2016). Especially in online learning environments students often have to operate autonomously, which makes the ability to self-regulate learning processes even more important (e.g., Wong et al., 2019; Jansen et al., 2020). Moreover, students need to be equipped with strategies to regulate their own learning and development throughout their lives [i.e., lifelong learning (European Commission/EACEA/Eurydice, 2015)] To self-regulate their learning students need to be able to accurately keep track of their own learning process (i.e., monitoring) and use that information to regulate their learning process [e.g., select appropriate learning tasks (Zimmerman, 2008; Bjork et al., 2013)].

Yet, studies have shown that SRL is difficult for students (e.g., Bjork et al., 2013) because they are not capable of accurately judging their own learning processes and use this judgment to regulate further learning (e.g., Dunning et al., 2003; Dunlosky and Lipko, 2007; Thiede et al., 2009). However, most students do not get instruction about how to study (Bjork et al., 2013) and students are largely unaware of learning strategies which could help them to study effectively (e.g., McCabe, 2011; Blasiman et al., 2017; Dirkx et al., 2019; Cervin-Ellqvist et al., 2020). Without instructional support, students often overestimate their understanding (Thiede et al., 2009) and memory of learning materials (Dunlosky and Lipko, 2007), which can have detrimental effects on subsequent learning activities (Dunlosky and Rawson, 2012), academic success, and their capacity to become life-long learners. Therefore, we developed a mobile application to support students SRL processes and provide them with information on how to use effective study strategies. In this article a theoretical background, description of the app’s features and design choices are presented. Also, data from the application in presented to give provide an idea of how the app has been used.

### Theoretical Background

Self-regulated learning (SRL) is the degree to which people are “metacognitively, and behaviorally active participants in their own learning process” (Zimmerman, 1989, p. 4). According to the model of SRL by Zimmerman (2008) there are three phases in SRL: the forethought, performance and reflection phase. In the forethought phase students prepare their learning session, for example, by analyzing the task and setting their goals. Then in the performance phase students monitor and control their learning and use strategies to execute the learning task. In the third phase, the reflection phase, students evaluate their learning session and reflect on it (e.g., satisfaction). In this model of SRL both cognitive and metacognitive processes take place. Metacognitive processes for example are, students setting learning goals, monitoring learning processes, and controlling their learning. Using study strategies during the performance phase entails all kinds of cognitive processes, such as elaboration or self-testing.

There have been numerous studies on supporting student’s cognitive and metacognitive activities to enhance learning processes and outcomes. Indeed, a meta-analysis by Dent and Koenka (2016) showed that cognitive strategies and SRL are significantly correlated to academic performance. Moreover, Dent and Koenka (2016) suggest that the metacognitive processes that allow students to self-regulate their learning and choose which cognitive strategies to use, may be more important than applying cognitive strategies. In other words, knowing what type of action to take in the learning process at what moment seems crucial. Also, research has shown that both prompting cognitive and metacognitive strategies is effective to support SRL (Devolder et al., 2012). Interventions to support SRL processes based on metacognitive theories, like metacognitive reflection (Dignath and Büttner, 2008) and planning strategies (Dignath et al., 2008), work well for students in secondary education and beyond. In addition, a recent review on writing journals as a promising tool for learning by Nückles et al. (2020) confirmed the benefits of combining cognitive and metacognitive prompts when supporting students during learning. Moreover, research has shown that the most optimal sequence of prompts consists of metacognitive prompts first followed by cognitive prompts (Roelle et al., 2017). Thus it seems promising to support students’ SRL processes by designing effective scaffolds in which both metacognitive and cognitive strategies are elicited in order for students to get the most out of it.

Yet, when supporting students, it is crucial to provide the right information at the right time (see Van Merriënboer et al., 2002). Indeed, several studies have shown that using daily diaries or interactive ambulatory assessments can provide important insights into students’ SRL behaviors (e.g., Fabriz et al., 2014; Wäschle et al., 2014; Liborius et al., 2019) and can even support SRL and subjective learning experiences (e.g., Loeffler et al., 2019; Broadbent et al., 2020). An interesting way to provide students access to scaffolds for their (self-regulated) learning processes at anytime and anywhere, is using mobile technology (e.g., Loeffler et al., 2019; Palalas and Wark, 2020). That is, almost every student has a mobile phone and with this mobile device supportive applications can be brought close to the student’s learning process at anytime and anywhere.

Using mobile technology to support learning or to create a learning environment is also called mobile learning (m-learning) and can be formal, informal or in a combination (Viberg et al., 2021). It was found to be related to study success in educational, non-educational as well as informal learning settings (e.g., Wu et al., 2012; Crompton and Burke, 2018; Shadiev et al., 2020). A recent review on the relationship between m-learning and SRL (Palalas and Wark, 2020) showed that m-learning enhanced SRL, and the other way around. One of the conclusions of the review was that because of the flexibility and portability of mobile technologies, they offer students the opportunity to exercise their agency and use their mobile device as a cognitive and metacognitive tool (Palalas and Wark, 2020). Therefore, mobile technology seems very suitable for supporting SRL.

For example, a study by Tabuenca et al. (2015) showed that tracking time during the learning process using mobile devices with graduate students had a positive effect on time management. In a study by Loeffler et al. (2019) it was found that providing prompts and feedback about metacognitive strategies during the preparations for a written exam using mobile technology, promoted metacognitive strategies, internal resource management and subjective learning experiences. Also, a study by Broadbent et al. (2020) replicated and extended a study by Bellhäuser et al. (2016) using a web-based SRL training and a mobile-app based diary to improve SRL. Specifically, the web-based SRL training provided students with information about the three phases of the Zimmerman SRL model (i.e., forethought, performance, and reflection) during three sessions which were spread across 21 days. In addition, on each of those 21 days students were prompted *via* the mobile app to answer whether they were planning to study that day and if so, what SRL strategies they were going to use and how they felt (positive or negative affect). Also, after studying, students were also prompted to report the strategies they had used and report on their affect. Broadbent et al. (2020) found positive effects in terms of resource management (i.e., time and space), metacognitive and cognitive strategies of using the domain-independent web-based SRL training module and a mobile-app in which students wrote short diary entries. Interestingly, the combination of the web-based training module and the mobile-app, was found to benefit the students’ use of SRL strategies the most. Moreover, using the mobile-app for daily diaries only did not seem to improve students’ SRL strategies compared to a control condition. The authors highlight that self-monitoring *via* a daily diary only, is probably not enough if someone does not know *how* to self-regulate his or her learning. Hence the combination of information on the three SRL phases with prompts at the beginning and ending of a study session seem to really support students to self-regulate their learning.

Extending these findings and exploring a more coherent way to scaffold both cognitive (i.e., study strategies) and metacognitive processes (i.e., planning and reflection) to improve SRL by students, we developed the Ace your self-study app (Study app in short^1^). In the Study app processes from the forethought, performance and reflection phase are prompted to support students’ SRL processes while engaging in self-study. Also, 20 evidence-based study strategies are offered with a short description and a video on how to use them (see Supplementary Appendix A). This combination of features provides the student with the information on how to self-regulate their learning using study strategies but also prompts them to plan, monitor and reflect on their own learning processes during self-study.

## Description of Ace Your Self-Study App Features

### Forethought Phase

**FIGURE 1:**
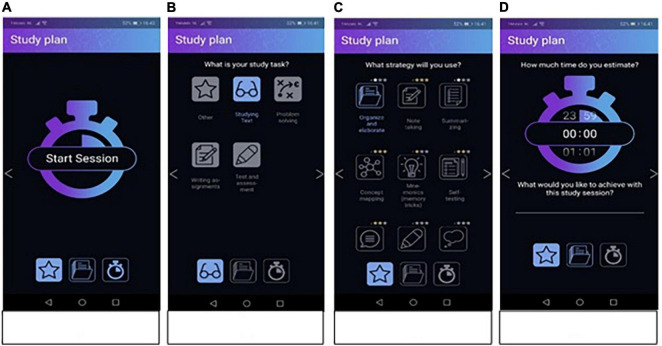
Screenshots from the forethought phase, panel **(A)** shows the first “study plan” screen to start a session, panel **(B)** shows the second “study plan” screen at which students choose the type of task, panel **(C)** shows the third “study plan” screen at which students choose a strategy. Panel **(D)** shows the fourth “study plan” screen at which students can set the time and fill out their goal.

In the forethought phase, when students open the app, they will start with making a study plan for the study session they are about to start (Figure 1). After clicking on “start session,” they are invited to choose the task they will be working on, that is, “studying text,” “solving problems,” “writing assignments,” “test and assessment,” or “other.” Based on this choice, a selection of study strategies will be shown. Offering this selection of study strategies is based on the idea that learning is a generative activity during which students actively construct meaning from the materials they are studying by reorganizing and integrating it into their already existing knowledge. This process is dependent on how students make sense of their learning materials, for example, by using learning or study strategies (Fiorella and Mayer, 2016). In addition, some strategies can be applied more effectively in certain learning contexts compared to others (Schunk, 2014; Fiorella and Mayer, 2016). Therefore, based on the learning context in which strategies were investigated or described in the research literature, we organized study strategies into the categories “studying text,” “solving problems,” “writing assignments,” and “test and assessment.” Just in case these categories would not suit the students’ aim of the study session, we included the category “other.”

If a student would choose “studying text” the following strategies would be highlighted as a suggestion for students: summarizing (e.g., Wittrock and Alesandrini, 1990; King, 1992; Gil et al., 2010), concept mapping (e.g., Nesbit and Adesope, 2006), organize and elaborate (e.g., McDaniel and Einstein, 1989; Wade, 1992; Mintzes et al., 1997), note taking (e.g., Barnett et al., 1981; Benton et al., 1993; Peverly et al., 2003), mnemonics (e.g., Wang and Thomas, 2000; Rummel et al., 2003; Soemer and Schwan, 2012; Ormrod, 2016), self-testing (e.g., Roediger and Karpicke, 2006; Hartwig and Dunlosky, 2012; Fiorella and Mayer, 2016), self-explaining (e.g., Chi et al., 1989; Renkl, 2002; Ainsworth and Th Loizou, 2003; Fiorella and Mayer, 2016), drawing (e.g., Fiorella and Mayer, 2016; Fiorella and Zhang, 2018), imagining (e.g., Fiorella and Mayer, 2016), spacing (e.g., Carpenter et al., 2012), and self-managing cognitive load (e.g. Roodenrys et al., 2012; Sithole et al., 2017; Eitel et al., 2020). If a student would choose “problem solving” the following strategies would be highlighted: generate and test (e.g., Schunk, 2014), analogical reasoning (e.g., Gick and Holyoak, 1980, 1983; Halpern et al., 1990), brainstorming (e.g., Mayer, 1992; Schunk, 2014), worked-out examples (e.g., Sweller et al., 1998, 2019; Van Gog and Rummel, 2010), self-testing, self-explaining, drawing, imagining, and self-managing cognitive load. If a student would choose “writing assignments” the following strategies would be highlighted: models for writing, clear writing goals, plan-draft-revise, and organize ideas for writing (e.g., Graham and Perin, 2007; Graham et al., 2013). If a student would choose “test and assessment” the following strategies would be highlighted: self-testing and expressive writing (e.g., Ramirez and Beilock, 2011). Students can select a strategy for this study session by clicking on it. They will get more information on the strategy including a text, an image and a short video on how to use the strategy. For an overview of the strategies per task type see Supplementary Appendix A. After selecting a strategy, students are asked to set the time for their study session in hours and minutes. In addition, they can choose to set a goal for their session (Figure 1).

### Performance Phase

**FIGURE 2:**
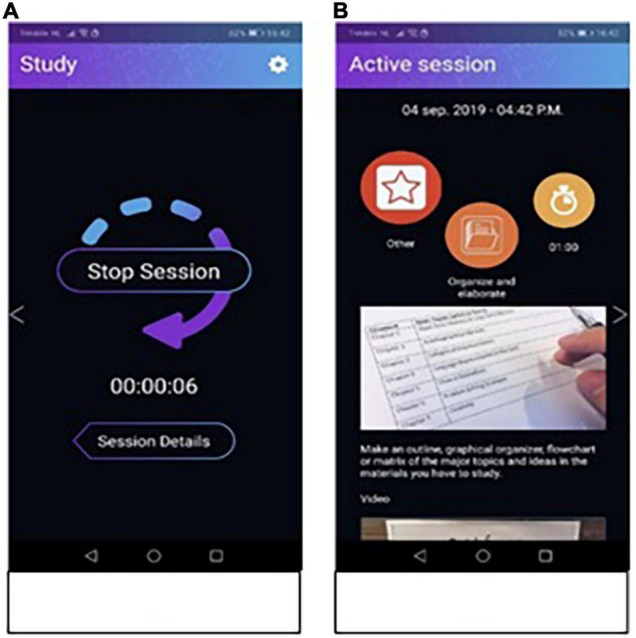
Screenshots from the performance phase. Panel **(A)** shows the defaults screen during the performance phase which shows a timer. Panel **(B)** shows the summary of the “study plan” made in the forethought phase.

After students have made their study plans in the forethought phase they start the actual study session in the performance phase. In this phase, there is little to see or do in the application itself, because it is considered important that the students do not work on their phones. Instead, they are only supposed to use the mobile application on their phone to help them plan, monitor and control their learning processes. Therefore, the only option students have during the performance phase other than reading their study task, is looking back at their study plan including information about the study strategy that was chosen (Figure 2).

### Reflection Phase

**FIGURE 3:**
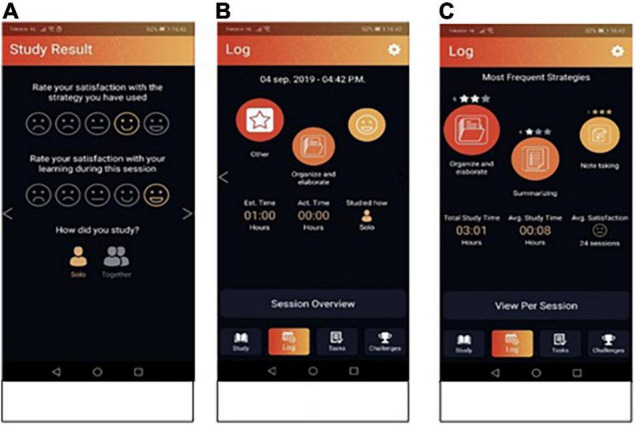
Screenshots from the reflection phase. Panel **(A)** shows the two ratings students have to fill out. Panel **(B)** shows the log for a single session. Panel **(C)** shows the log across sessions.

When students decide to stop their study session, they enter the reflection phase, in which they are prompted to reflect on the result of their study session (Figure 3). They are asked to rate their satisfaction with the study strategy they have used and with their learning during the session using a 5-point rating scale with smileys. Also, students were asked to indicate whether they had studied alone or together with other students. Note, this feature only allows capturing this information for the log files for the purpose of reflection on the learning process. There are no other features in the app that support social interaction through the app in the current version. After providing these ratings, students can use the log to look at the summary of their session or a summary across multiple sessions. These logs provide them with information on the strategies, ratings, studying alone or together and time they have planned and actually spent. That way, the app can support the reflection phase in SRL.

### Gamification Elements

**FIGURE 4:**
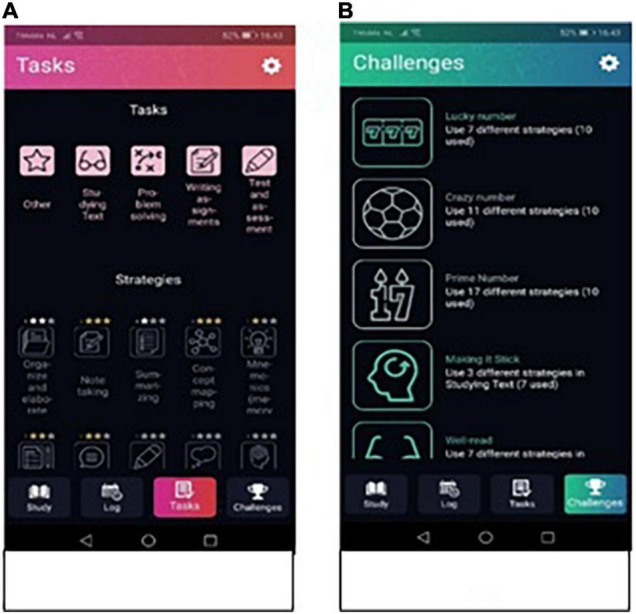
Screenshots of gamification elements in app. Panel **(A)** shows the overview of the strategies with the level of use depicted in stars. Panel **(B)** shows the challenges students can take when using the app.

Research has shown that gamification elements such a as levels, points and scoreboards, can increase student motivation and performance. Gamification elements provide clear goals and rewards for students which keeps them engaged and motivated (Su and Cheng, 2015; Mekler et al., 2017). Therefore, both in the tab “Tasks” and the tab “Challenges” some gamification elements were implemented in the app. In Tasks students can find all the types of tasks and all the strategies (Figure 4). Here the student can also see how many stars (i.e., levels) per strategy are earned already. The last tab Challenges provides the student with some challenges in terms of planning sessions and using a variety of learning strategies. For example, “Lucky number, use 7 different strategies.” Both the stars and the challenges are gamified elements to stimulate the users to use the app and the strategies in the app to its full potential for learning. All the challenges are provided in Supplementary Appendix B.

## Conceptual Design and Design Process

The intention behind the conceptual design was to create a streamlined user experience with the least amount of friction caused by “trying to figure out the app.” Two design principles were selected by the designer to guide the conceptual design of the current app (Lidwell et al., 2010). The first is the 20/80 rule (a.k.a. Pareto principle) which governs how a few critical features create the most significant effect. The sizeable stand-alone “*start session*” button found on the app’s main screen represents this concept. In this case students can clearly only choose to click “start session” (Figure 1A) which is a significant choice. The second is the Flexibility–Usability Tradeoff principle, which states that when flexibility increases usability and performance decrease. The “wizard” or linear session set-up, which allows students to create a step-by-step study plan, is an example of this concept. Both of these principles were used as guidelines to design the Study app.

The app follows a design-driven UX (User Experience) approach to development, including the co-design and creation with researchers, students, and developers. The purpose of a design-driven approach is to select technology for the best impact and avoid the typical pitfall of “we want an app” syndrome (e.g., using the technology simply because it is available). That is, the app was specifically designed to create the best impact for its purpose. The design process started with an analysis phase to define the purpose of the app and followed the exploratory investigation of paradigms used in apps popular with the target audience. The approach to deciding the interaction and app flow started with a diagram that documented the architecture. From the initial sketch, a wireframe of each screen was mocked up with a preliminary positioning of interactive elements (e.g., buttons). Using Adobe XD software, a basic interactive mock-up called a click-through was created based on the wireframes and architectural flow. The click-through was then black-box tested, that is, given to co-designers to explore without explanation for usability and usefulness for the research goals. The resulting feedback from the user tests was then used to improve interaction. The next click-through version included a visual aesthetics (e.g., colors, icons, fonts, etc.) upgrade, which was then tree tested by the target audience. The tree testing method was used to determine if the target audience could navigate and discover the core functionality of the app. Feedback from the play-test was used to again iterate on the visual design and interaction design. At this point, the design hypothesis was considered solid enough to begin the development of the app. Native iOS and Android programming languages were used to develop the app for deployment to smartphones and tablets. Additionally, a CMS (content management system) was created to allow researchers to add and edit content and manage user data.

### Gamification Design Process

Gamification is the term used to describe the application of game principles and patterns to motivate users to accomplish daily activities. The aim is to drive user activity by closing or tightening the feedback loop (e.g., scoring points) and allow users a way to track their progression (e.g., achieving a high score). Game principles also include the use of player communities to create competition, cooperation, peer-pressure, or social connectivity. The app’s gamification aimed to encourage students to explore different types of study strategies and adhere to studying with the app. Two kinds of gamification elements are used to accomplish these aims. *Challenges* are intended to stimulate students to explore different types of study strategies. Users can find a list of challenges they try to fulfill by using the Study app. For example, the challenge “Lucky number” states: “Use 7 different strategies” (see Supplementary Appendix B for an overview of the challenges). When students finish a challenge, the challenge will be highlighted in their list of challenges. *Stars* allow the user to track their use of a single study strategy. For each instance a study strategy is used, the next level will be reached which will then be indicated by a star depicted with the strategy name (see Figure 4). A maximum of three stars can be earned.

The gamification design process began with setting the design goals, followed by a pitch to the project researchers of game elements that could accomplish these goals. From the concept pitch, there was a brainstorm session with students to gather ideas on how they would be best motivated to use the app. The result of these initial activities were ideas for a *star* system, a *challenge* system, and a *cooperative user-sharing* system. However, due to project constraints, not all these features could be built. Eventually, a decision was made to implement the *star* and *challenge* systems. Lastly, a usability black-box user test was done to determine if users and stakeholders understood the gamification.

## System Architecture

The system architecture includes a CMS accompanied by a public website and two apps (iOS and Android). The system architecture facilitates researchers with features for managing content, moderating users, exporting data, and website customization.

The CMS is a back-end interface and website built using open-source software and hosted on a LAMP (Linux-Apache-MariaDB-PHP) server. The relational database on the server stores all log records. Communication with the app occurs through a RESTful API (Application Programming Interface). All connections and webpages of both the CMS and website are encrypted using SSL (Secure Sockets Layer). More information about the source code is available upon reasonable request.

Administrators of the CMS can assign different roles or access rights to different CMS users, including the moderation of the study tasks, strategies and challenges available in the app, insight into sessions and account data, and customization of the webpage content. The CMS provides researchers with various functionality that includes adding and editing content, moderating users, exporting research data, and editing the public website content. A researcher can add or edit the tasks, strategies, and challenges to the app without technical support. Students (end-users) encounter these changes to the content when online and after restarting the app. The system determines by equal distribution if a student will have a gamified or non-gamified version of the app. Researchers can also manually set a student’s app to gamified or non-gamified. Furthermore, user account and session data can be exported as CSV or TAB-delimited files for research purposes. Session data exports include the following:

**Table:** 

Account:	User ID, date of birth, and gender.
Current type:	Indicates if the app used during the session is gamified or standard.
Task:	The type of study task done during the session.
Strategy:	The type of study strategy selected for the session.
Goal:	The study session goal that was entered by the student.
Estimated time:	The student’s estimated time (minutes) for the study session.
Actual time:	The student’s actual time (seconds) of the study session.
Study session rating:	Indicates on a scale (1–5) the student’s satisfaction with the strategy and learning; and if the session was alone or partnered.
Start:	Provides a timestamp for the beginning of the session.
Stop:	Provides a timestamp for the end of the session.
Sync:	Provides a timestamp for when the user’s data was synchronized with the database.

The implementation of the system architecture took into account the need to include future functionality, for example, including a feature for language localization, connecting to an LMS (learning management system), and more in-app questionnaires.

The app is built with SWIFT (for iOS) and Java/XML (for Android), while a local SQLite database is implemented for each app installed on a specific device. The rights to the source code for the apps belong to the developer. Students are required to create an account with a valid email address and a password in order to be able to use the app. A verification email is sent to the supplied email address on account creation. The user has to click on a link in this verification email before logging on and starting using the app. The registration process also includes collecting research data regarding the students’ year of birth and gender. The local SQLite database is used to store a duplicate of all task, strategy, and challenge data needed for the use of the app, which allows the app to be used even when a user is not online. All study sessions are stored locally and uploaded to the CMS when an offline user goes online again. This setup also allows for migrating an account to a different device or use of the app by the same user on multiple devices, such as smartphones and tablets.

The process to develop the CMS, website, and apps included an initial evaluation of the available technology. During the evaluation, considerations relevant to the project’s needs were determined. For example, other development frameworks may allow for publishing for iOS and Android from a single code base but may not allow essential features such as push notifications. After the evaluation, it was decided to develop the apps natively, i.e., create two separate code bases in the native programming languages of Android and iOS. Android was chosen to be developed first because of the developer’s familiarity with JAVA/XML and the ease with which the app could be tested on Android devices. When developing for Android, the developer can build an APK and distribute it by several means to be installed on a device for testing. In contrast, iOS requires users to install an app that manages the installation and testing of apps. The CMS and website were developed simultaneously during the development process, while the iOS version was developed last.

**FIGURE 5:**
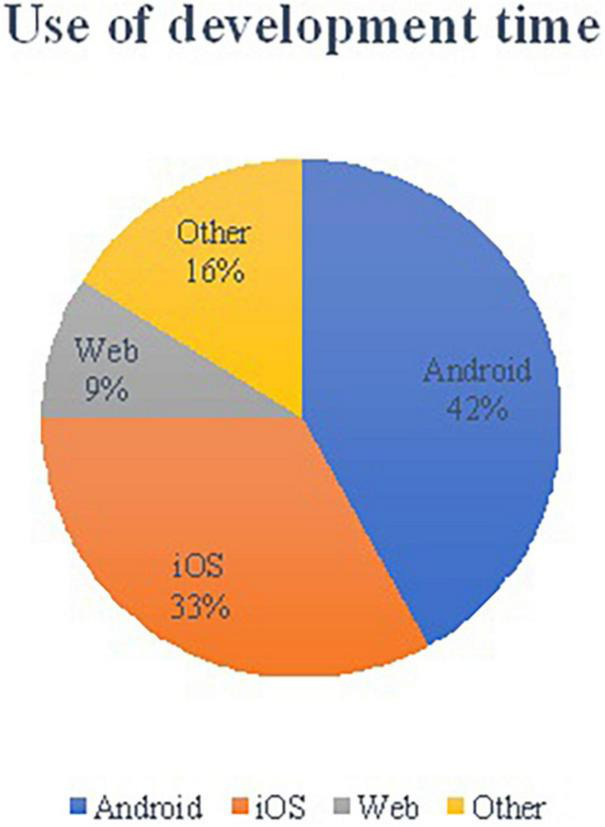
Use of development time.

Building natively in iOS and Android means two separate apps need to be developed. Six hundred and fifty hours were used to develop the initial system. Figure 5 provides the percentage of time needed to develop all the aspects of the system. There was a slight gain in efficiency for the developer by being familiar with the app’s interaction design when developing the app again for iOS.

Google Play and Apple Store are distribution platforms needed to distribute apps to students (end-users) efficiently. When submitting to these stores, additional development time may be needed for minor changes to the app to meet the standards and criteria established by the distribution platforms.

Once an app is available through a distribution platform, it does not guarantee that it will continue to function. For example, updates to operating systems will eventually make an app obsolete and no longer run on a device. For this reason, an SLA (service level agreement) is created with the developer. The purpose of the SLA is to ensure that the app is managed and maintained to keep the app functional. Furthermore, it determines how a developer prioritizes solving issues, helping end-users, managing the stores, and making minor improvements.

In summary, our guidelines for developing an app system architecture would be as follows:

• Evaluate the available technology; and thoroughly understand the trade-offs of each available platform.
• Work as a multi-disciplinary team.
• Design and develop iteratively.
• Include an interaction designer that can bridge research, psychology, computer science, and human-centered design.
• Build and test regularly.

## Organizational Context

In Marc Prensky, (2001, p.2) wrote: *“Our students have changed radically. Today’s students are no longer the people our educational system was designed to teach”*.

Stating that the students had become digital natives with a high level of understanding the digital language and the educators are digital immigrants speaking an outdated language, his words have an even greater value today. In this day and age our students have changed even more and are matured digital natives. Smartphones, laptops, and tablets are mainstream devices and present not only in our students daily life but also in our educators daily life. Fortunately, the gap between students and educators when it comes to being a digital native is not as large or definite as some might suggest (Helsper and Eynon, 2010). Breadth of use, experience and educational levels also play a role in having advanced interaction with the internet. Moreover, it is possible for adults to become digital natives. Hence, if used the right way, smartphones and tablets can act as engaging platforms to help educators to immerse these students into educational content.

Back to Prensky, the same statement can be made for universities. The primary task of a university is not to design, develop, and deploy new educational technologies. It is a fact that IT projects are notorious for running late, being over budget and failing on all levels (Williams, 2017). Designing, developing, and deploying mobile applications within a university context is an even more costly and time consuming process. The life cycle for an app development starts with picturing the entire range of stages and procedures to go through. Next to designing, developing, and deploying the app all parties involved need to take several things in consideration in the implementation phase.

Firstly, teams might encounter several legal questions in regards to privacy issues and intellectual property rights. In regards to privacy issues (mostly concerning the GDPR) the data that is collected from the data subjects contribute to the underlying goals of the research. GDPR-proofing the application also includes a full privacy statement, an End Users License Agreement (EULA) and general terms and conditions for usage. To check whether an application is GDPR proof it is important to check the following:

- Determining the data subject.
- Determine the goal and purpose of storing the data of the data subject.
- Determine if sensitive personal data of the “data subject” is being requested/stored?
- Determine if personal data is being requested/stored.
- Determine if a combination of “general data” can lead to a (in)direct identification of the data subject.
- Determine which party is the “data controller.”
- Determine if the app is working with “data processors,” if so, identify the data processors.
- Determine the duration of the data storage.
- Determine the storage location of the data.
- Determine the method of removing personal data in order to comply with the right to be forgotten.
- Determine a plan of action in case of a data breach.
- Determine how consent for data processing is obtained.
- Determine if a processor agreement is necessary.

Most of these points are covered by the universities privacy policy, however, the importance of safeguarding personal data cannot be understated.

### Intellectual Property Rights

In this specific case we have developed a mobile application, which is a software application designed to run on a mobile device, i.e., a smartphone. To protect applications from infringement by third parties it is eminent to determine the ownership of the application. In general software applications such as the Ace your self-study app can be protected by several intellectual property rights. The most obvious questions related to intellectual property rights are:

### Patent

A patent is usually obtained to protect technical inventions that are novel. In this specific case obtaining a patent for the app would be a lost cause. The app is an obvious next step in the advancement of technology.

### Trademark

Due to the highly competitive nature of the industry the protection of the name, logo, patterns, shapes, colors and other characteristics that distinguishes the application from other available applications on the market can be obtained by registering a trademark.

### Copyrights

All mobile applications are software applications designed with a unique source code that allows it to run on a specific device. Due to the unique composition of every code written, it meets the standards of copyright protection.

### Design Protection

This guarantees the project team the exclusive rights to use the design and to protect the appearance of the application or parts of it, including contours, colors, and shapes.

Secondly, it is recommended to draft a Service Level Agreement with an independent trusted third party. Most universities do not have the capacity to maintain and update the licenses needed for the application. This can be circumvented by hiring a third party. Considering the fact that most development teams only calculate a sufficient budget for the development, it is highly recommended to have a healthy budget in place for new releases, hosting, maintenance, security, software updates, and app store licenses.

Last but not least, connecting research to a mobile application is highly risky undertaking. The research can only be conducted as long as the application is running.

## Data From the Ace Your Self-Study App

In September 2021, 4,254 accounts were registered for the Ace your self-study app between December 2017 and September 2021. Most users were presumably invited to use the app by their teachers or trainers in higher education settings as the app was presented at several national and international meetings and conferences on educational innovations, teaching and learning for researchers and educational professionals (e.g., EARLI conference, SURF education days). It is also possible for learners to have found out about the app by themselves, *via* conferences they attended or because it is freely available in the App and Play Store and they found it there. From the persons who registered for an account 1,134 indicated they are male and 3,120 indicated they are female. Their mean age in years is 24 years (SD = 9.05). The most frequent age was 20 years old. These users have completed 6,505 study sessions in total. To provide an idea about what these study sessions looked like, we will present data on the number of sessions, the duration of sessions, the strategy choices, the satisfaction with the strategy that was chosen and the satisfaction with learning during the self-study session in general.

### Number of Sessions

**FIGURE 6:**
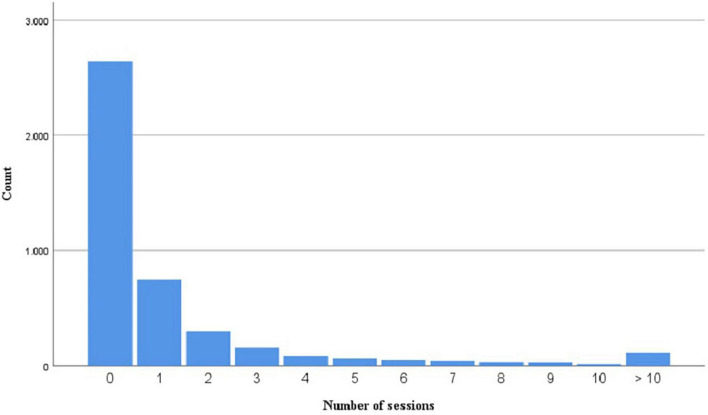
The count of users with a certain number of sessions.

Figure 6 shows that very often users did not create a study session but most likely just explored the app (*n* = 2,643). Many users choose to have 1 study session (*n* = 745). Fewer users had 2 (*n* = 300), 3 (*n* = 155), 4 (*n* = 84), 5 (*n* = 62), or more sessions. As the data are skewed, we used a Mann–Whitney U test to explore differences in the number of sessions by gender. No differences in the number of sessions between males (*Mdn* = 0) and females (*Mdn* = 0) were found, *U*(*N*_*males*_ = 1,134, *N*_*females*_ = 3,120) = 1,822,898.00, *z* = 1,822,898.00, *p* = 0.080. In addition, year of birth was not significantly correlated to the number of sessions participants had, *r* = 0.028, *p* = 0.065.

### Duration of Sessions

**FIGURE 7:**
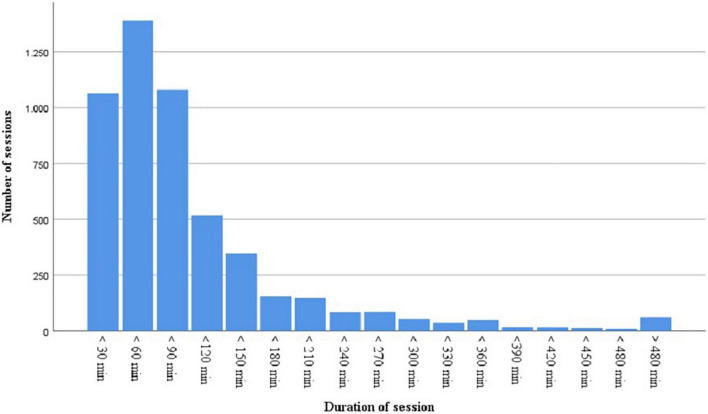
The number of sessions of a certain duration.

To get an idea of the duration of valid study sessions, we selected the sessions that lasted from 1 min up to 12 h (*n* = 5,597). Sessions that were shorter than 1 min (*n* = 626) or longer than 12 h (*n* = 290), were not considered here. For sessions shorter than 1 min it seems highly unlikely a user would have had the chance to set up a study session and for sessions longer than 12 h it is very likely a user forgot to stop the study session. Most sessions lasted between 30 and 60 min (*n* = 1,398), followed by 30 min or less (*n* = 1,065), and between 60 and 90 min (*n* = 1,081). There are also quite some sessions of 2 h (*n* = 518), 2.5 h (*n* = 346), 3 h (*n* = 155), and 3.5 h (*n* = 147). Only 8% of the sessions (*n* = 428) lasted 4 h or longer (see Figure 7). As the data are skewed, we used a Mann–Whitney U test to explore differences between male and female users in the duration of sessions. It was found that the duration of sessions was significantly different for males (*Mdn* = 59.43) compared to females (*Mdn* = 60.94), that is, females were found to have a slightly longer duration of their sessions, *U*(*N*_*males*_ = 1,183, *N*_*females*_ = 4,414) = 25,751,397.00, *z* = 2,751,397.00, *p* = 0.004. Note that because of the high number of sessions, a small difference in the duration of the sessions, reached significance. In addition, age was not significantly correlated to the duration of sessions, *r* = 0.021, *p* = 0.118.

### Study Strategies

**FIGURE 8:**
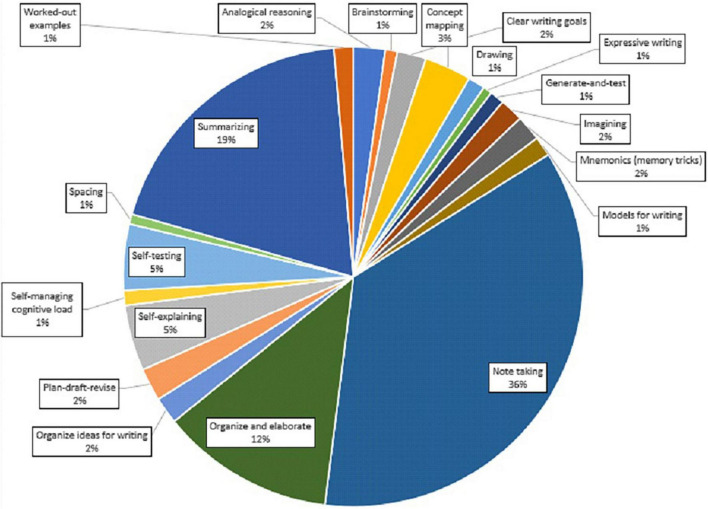
A pie chart showing the percentage for each strategy’ in the total of strategy’ choices.

As shown in the pie chart below, all kinds of strategies were chosen by the users. There are 20 different strategies in the chart. Notetaking was chosen most often (36%), followed by summarizing (19%), organize and elaborate (12%), self-testing (5%), self-explaining (5%), and concept mapping (3%). For the remaining strategies the percentages are small (only 1–3%, see Figure 8).

### Satisfaction With Strategies and Learning

Users (*N* = 1,246) were quite satisfied with the strategy they had chosen during their study sessions. On a 5-point scale the users indicated 3.44 on average (SD = 1.50) as their satisfaction score with the strategies they had chosen. As the data are skewed, we used a Mann–Whitney U test to explore the effect of gender on the satisfaction with the strategy. No difference in the satisfaction with strategies was found between males (*Mdn* = 4.00) and females (*Mdn* = 4.00), *U*(*N*_*males*_ = 303, *N*_*females*_ = 943) = 148,774.50, *z* = 148,774.50, *p* = 0.271. In addition, the age of the users was significantly correlated to the satisfaction with strategies, *r* = −0.152, *p* < 0.001. This seems to suggest that the older the users were the less satisfied they were with the strategies they had used.

The users satisfaction with their learning during the study session was slightly, 3.33 (SD = 1.50) on a 5-point scale, lower but still moderate. As the data is skewed, we used a Mann–Whitney U test to explore the effect of gender on the satisfaction with learning. No difference was found in the satisfaction with learning between males (*Mdn* = 4.00) and females (*Mdn* = 4.00), *U*(*N*_*males*_ = 303, *N*_*females*_ = 943) = 140,244.00, *z* = 140,244.00, *p* = 0.626. In addition, the age of the users was significantly correlated to the satisfaction with strategies, *r* = −0.138, *p* < 0.001. This seems to suggest that the older the users were, the less satisfied with their learning they were.

### Usability

A group of 45 college students (*M_age_* = 20.84, 39 females and 6 males) in an undergraduate psychology program used the Study app for one self-study session to test the usability of the app. They used the Study app in a 60 min self-study phase during which students studied a scientific article. At the end of the session, students answered four questions to evaluate the use of the Study app (see Supplementary Appendix C). As shown in Table 1, the Study app was evaluated quite positively with 5.69 out of 7 points on average. Specifically, students rated the app as quite easy to understand, easy to navigate, intuitive to use, and the strategies to be clearly described.

**TABLE 1:** Descriptive statistics.

	Mean (SD)	Minimum	Maximum
Evaluation questions (total)	5.69 (0.88)	2.25	7
Easy to understand strategies	5.80 (0.99)	2	7
Clearly described strategies	5.78 (1.00)	3	7
Easy to navigate app	5.71 (1.04)	3	7
Intuitive to use app	5.47 (1.01)	2	7

## Discussion and Conclusion

Especially in online learning environments the ability to self-regulate learning processes is important to learn effectively in an autonomous or independent way (e.g., Wong et al., 2019). Yet, many studies have shown that SRL, that is, effectively monitoring and regulating one’s own learning processes, is difficult for students (e.g., Dunning et al., 2003; Dunlosky and Lipko, 2007; Thiede et al., 2009). This means there is a need for support and instruction on how to self-regulate learning and use study strategies. However, most students do not get this support or instruction about how to study (Bjork et al., 2013). In addition, most students are unaware of learning strategies which could help them to study effectively (McCabe, 2011; Dirkx et al., 2019). This is problematic as it was found that without instructional support, students often overestimate their learning processes (e.g., Dunlosky and Lipko, 2007; Thiede et al., 2009) and prematurely stop studying (Dunlosky and Rawson, 2012). Therefore, we developed a mobile application to support students’ SRL processes and provide them with information on how to use effective study strategies.

To accommodate the often autonomous learning situation of students in higher education which could take place anytime or anywhere, we have used mobile technology to create an application to support self-study activities, the Ace your self-study app (Study app). In the Study app processes from the forethought, performance, and reflection phase based on the model of SRL by Zimmerman (1989, 2008) are prompted to support student’s SRL processes while studying. Next to these phases, 20 evidence-based study strategies are offered with an explanation on how to use them. Because gamification elements such as levels, points and scoreboards, can increase student motivation and performance (Su and Cheng, 2015; Mekler et al., 2017), some gamification elements were implemented in the app. Students can earn stars (i.e., levels) per strategy and they are challenged in terms of planning sessions and using a variety of learning strategies.

The conceptual design was chosen to create a streamlined user experience with the least amount of friction caused by “trying to figure out the app.” The app follows a design-driven UX approach to development, in which the co-design and creation with researchers, students, and developers is central. The development of the mobile application followed an iterative design, built, test and evaluate cycle in which all stakeholders were involved. Next to the development and design of the Study app, several legal questions about privacy issues and intellectual property rights are important. With regards to privacy issues, the data that is collected from the data subjects contribute to the underlying goals of the research. Therefore, GDPR-proofing the application also included a full privacy statement, an EULA and general terms and conditions for usage. Also, a Service Level Agreement with an independent trusted third party to maintain and update the licenses needed for the application was created. This is particularly of importance when considering future research plans involving the usage of the app.

Looking at the data, very often users did not create a study session but most likely just explored the app. Of the users who started a session, most users chose to have one study session and fewer users had two or more sessions. The fact that only 1,246 out of 4,254 registered accounts had study sessions, is a remarkable finding. Potentially this could be the case because of a mismatch between the user’s needs and what the Study app offered. That is, the Study app was developed to support SRL activities during self-study sessions. Yet, research has shown that people often overestimate their learning (e.g., Bjork et al., 2013) and know little about study strategies (e.g., McCabe, 2011) that can help them to learn more effectively. Hence, perhaps potential users thought they did not need an app to help them regulate their learning and use effective study strategies during self-study. Future research could look into the experiences of persons who have used the app for self-study and those who have looked at the app but decided not to use it. Moreover, it would be interesting to investigate if applications that provide more guidance instead of leaving it up to the user, would have a different effect on user behavior. For example, a mobile application could also include push messages to provide suggestions or feedback with SRL activities. In addition, integrating the Study app into educational programs could allow for teachers or trainers to guide their students when it comes to using the app and the SRL support within the app to their benefit.

Based on the data from active users, we found that most sessions lasted between 30 and 60 min, followed by 30 min or less, and between 60 and 90 min. In a total of 6,505 study sessions notetaking was chosen most often (36%), followed by summarizing (19%), organize and elaborate (12%), self-testing (5%), self-explaining (5%), and concept mapping (3%). Users were quite satisfied with their strategy choices and learning in general during the sessions. Also, from the pilot study in which a small group of students used the Study app to study a scientific article, we found that students were generally satisfied with the app. They evaluated the Study app on different levels such as easy to understand, clarity of the strategies, easy to navigate the app and intuitive to use the app and scored moderately high on these aspects. However, this was a first pilot study and did not involve students actual study tasks at that moment. Therefore, future research could investigate a more ecological valid study situation in which students use the app for their self-study activities related to the courses they are taking. A first study in a more ecological setting has recently been carried out with first year psychology students during their first course (Baars et al., 2022). In the study of Baars et al. (2022) students were invited to use the Study app during their self-study sessions. The use of the study app was investigated in relation to motivation and SRL across the course. Results showed a significant increase in motivation and SRL across the 5-week course but this was not related to Study app use during the course. Yet, most students used the app only for a limited number of self-study sessions. As this was a correlational study, it is hard to conclude anything about the effect of the app. Future research could apply randomized controlled trial (RCT) studies to investigate the effect of the app on SRL. Moreover, in terms of generalizability and validity, it would be valuable to investigate the use of the Study app in other fields besides psychology and other levels of education (e.g., secondary education) as well.

Although the Study app made use of several gamification elements (i.e., levels and challenges), it might not have been enough to affect the users. Possibly students can “game the system” by selecting strategies that could help them earn stars and finish challenges without actually using these strategies during their self-study session. After all, using the study strategies is something that happens outside the app (e.g., on paper or pc). Of course, if this happens, the app will most likely not support the regulation of the learning process during self-study sessions. Another limitation on gamification in the app was that there were no options for social interaction within the app. Options for users to share experiences or accomplishments in terms of self-study and using study strategies might be an interesting way to add social interaction as a form of gamification to the app (Sailer and Homner, 2020). Future research could look into the benefits of more social interaction and gamification on self-study effectiveness in terms of cognition, motivational and behavioral change.

The development of the Ace your self-study app and the results from the pilot study can provide valuable input for a discussion on applying theoretical knowledge to develop tools to support SRL. That is, the development of the app provides an example of a more holistic approach to supporting self-study sessions combining both cognitive and metacognitive strategies within the cycle of SRL proposed by Zimmerman (2008). As a practical implication, the app could provide teachers and students with a tool that provides evidence-based support for SRL processes during self-study. Yet, the holistic approach in the app based on all three phases of SRL including study strategies, could also cause limitations to researching the effect of the app. Namely, it complicates investigating the effect of the different aspects of the support that is offered in the Study app and differentiating which part would be causing what effect on SRL. Future research should, therefore not only focus on the effect of the app as a whole, but also on disentangling the contributions of the different aspects of support.

In sum, to support students’ self-study activities for them to effectively self-regulate their learning processes, a mobile application called the Ace your self-study app was developed. The choices involved in developing and designing the application were described in the current manuscript in which we presented the mobile application, the current state of use and pilot results on usability. In doing this we included the information and perspectives of the multidisciplinary team that worked on creating the Study app. Future research could investigate the effectiveness of the Study app with different types of self-study activities, educational levels, and study designs (e.g., randomized controlled trials) to provide more insight into using a mobile application with gamification elements to support SRL processes.

## Data Availability Statement

The raw data supporting the conclusions of this article will be made available by the authors, without undue reservation.

## Ethics Statement

Ethical review and approval was not required for the study on human participants in accordance with the local legislation and institutional requirements. The patients/participants provided their written informed consent to participate in this study.

## Author Contributions

MB, FZ, MH, EJ, and FP contributed to conception and design of the application. MB, FZ, MH, and EJ organized the database. MB performed the statistical analysis and wrote the first draft of the manuscript. FZ, MH, and EJ wrote sections of the manuscript. All authors contributed to manuscript revision, read, and approved the submitted version.

## Conflict of Interest

EJ was employed by Dev66. The remaining authors declare that the research was conducted in the absence of any commercial or financial relationships that could be construed as a potential conflict of interest.

## Publisher’s Note

All claims expressed in this article are solely those of the authors and do not necessarily represent those of their affiliated organizations, or those of the publisher, the editors and the reviewers. Any product that may be evaluated in this article, or claim that may be made by its manufacturer, is not guaranteed or endorsed by the publisher.

## Footnotes

1
www.aceyourselfstudy.nl
