# Supplementary material for: Ace Your Self-Study: A Mobile Application to Support Self-Regulated Learning
Source: Front Psychol. 2022 May 3;13:793042. doi: 10.3389/fpsyg.2022.793042 (PMC9110801; doi:10.3389/fpsyg.2022.793042)
Supplement: Supplementary file 1 [file Data_Sheet_1.docx]

Supplementary Material

**Appendix A**

**Study strategies organized by type of task**

| **Type of task** | **Strategy** | **Description** |
| --- | --- | --- |
| **Studying text** | Organize and elaborate | Make an outline, graphical organizer, flowchart or matrix of the major topics and ideas in the materials you have to study. |
|  | Note taking | Take notes about the main ideas and supporting details from the text in a notebook to keep paying attention to the materials you are studying. |
|  | Summarizing | Make a summary of the most important topics in the text. |
|  | Concept mapping | Make a concept map in which the most important concepts from the text placed in boxes which are connected to each other using lines to show the relation between concepts. |
|  | Mnemonics (memory tricks) | Create memory tricks to remember the materials you are studying. For example, find associated words, associated images, a rhyme or story which relates to the materials to help you remember. |
|  | Self-testing | Give yourself a practice test, to test your own understanding. |
|  | Self-Explaining | Explain in your own words the main ideas from the text. |
|  | Drawing | Make a drawing that depicts the text. |
|  | Imagining | Imagine a drawing that depicts the text. |
|  | Spacing | Spread repetitions of the learning content over time. Instead of studying long and intensively but only once, repeat learning content. For example repeat content from last week or the week before. |
|  |  |  |
| **Problem solving assignments (e.g. math, economics, statistics)** | Generate-and-test | If you have a problem or assignment with a limited number of solutions, generate the possible solutions and test them. If the first solution does not work, move on to the next most likely solution to the problem and so on. |
|  | Analogical reasoning | Find an analogy between the problem or assignment you are working on and a familiar situation. This familiar situation can help you to find the solution to the problem you are working on. Make sure the analogy is really similar in its structure to the problem or assignment you are working on. Only the details should differ. |
|  | Brainstorming | First, define the problem or assignment you are working on. Second, formulate possible solutions. Third, decide on criteria for judging the solutions you have generated. Fourth, use these criteria to select the best solution. Do not be too critical in the second step, keep room for creativity. |
|  | Worked-out examples | Find a worked-out example in which the solution path (steps) and the solution to the problem or assignment you are working on is shown. You can study the worked-out example to learn how to solve the problem or assignment you are working on. |
|  | Self-testing | Use a similar problem solving task to test your own understanding. |
|  | Self-Explaining | Explain in your own words how to solve the problem you are working on. |
|  | Drawing | Make a drawing that depicts the problem or the assignment. |
|  | Imagining | Imagine a drawing that depicts the problem or the assignment. |
|  |  |  |
| **Writing assignments** | Organize ideas for writing | Before you start writing, locate the information you need, generate writing ides, and organize them. You can use an outline, graphical organizer, flowchart or matrix to do so. |
|  | Models for writing | Find a model text of the type of text you are planning to write. Carefully analyze the critical elements in order to be able to use these elements or techniques from the model text in your own text. Note, use it as an example, do not plagiarize! |
|  | Clear writing goals | State specific goals about the written product you are about to write or about the revision you are about to make to a written product. Specify what you are planning to write or how you are planning to revise. |
|  | Plan-draft-revise | Work in cycles of planning what you want to write, making a draft of that and revise your writing. Repeat these cycles a couple of times. Note, you can do this together with other students. |
|  |  |  |
| **Test anxiety** | Write about your worries | Write down your exam related thought right before your exam. Writing may elevate the burden of your worries about your exam on your memory and help you perform better. |
|  |  |  |
| **Other** | *All of the above strategies can be chosen* |  |

**Appendix B**

**Challenges**

| Name | Description |
| --- | --- |
| \| Lucky number \| \| --- \| \| Crazy number \| \| Crime number \| \| Making it stick \| \| Well-read \| \| Librarian \| \| For research purposes \| \| Resolution for everything \| \| What are boundaries \| \| The hungry catapillar \| \| Isn't it poetic? \| \| Shakespeare \| \| Tiem trial \| \| Streak \| \| Streak \| \| Streak \| \| That's nice honey \| \| Blurred lines \| \| Michelangelo \| \| Rock star \| \| Know-it-all \| \| Are you Pondering what I'm Pondering \| | \| Use 7 different strategies \| \| --- \| \| Use 11 different strategies \| \| Use 17 different strategies \| \| Use 3 different strategies in Studying Text \| \| Use 7 different strategies in Studying Text \| \| Use 10 different strategies in Studying Text \| \| Use 2 different strategies in Problem solving \| \| Use 4 different strategies in Problem solving \| \| Use 7 different strategies in Problem solving \| \| Use 2 different strategies in Writing assignments \| \| Use 3 different strategies in Writing assignments \| \| Use 4 different strategies in Writing assignments \| \| Finish a 1-hour session \| \| Finish at least one session 3 days in a row \| \| Finish at least one session 5 days in a row \| \| Finish at least one session 7 days in a row \| \| Use the Drawing strategy 1 times \| \| Use the Drawing strategy 3 times \| \| Use the Drawing strategy 7 times \| \| Get 3 stars on 3 different strategies \| \| Have a total study time of 50 hours \| \| Finish a Brainstorming session with another person 3 times \| |

**Appendix C**

**Study app evaluation questions**

The following statements are about your experience with the Ace your self-study app. Please indicate to what degree you agree with the following statements on a scale from 1) *strongly disagree*, 2) *disagree*, 3) *somewhat disagree*, 4) *neither agree nor disagree*, 5) *somewhat agree*, 6) *agree*, to 7) s*trongly agree*.

|  |  |  |  |  |  |  |  |
| --- | --- | --- | --- | --- | --- | --- | --- |
| I found that the strategies provided by the Ace your self-study app were easy to understand | 1 | 2 | 3 | 4 | 5 | 6 | 7 |
| I found that the strategies provided by the Ace your self-study app were clearly described | 1 | 2 | 3 | 4 | 5 | 6 | 7 |
| I found the Ace your self-study app easy to navigate | 1 | 2 | 3 | 4 | 5 | 6 | 7 |
| I found the Ace your self-study app intuitive to use | 1 | 2 | 3 | 4 | 5 | 6 | 7 |
